# Assessment of the potential risk of leaching pesticides in agricultural soils: study case Tibasosa, Boyacá, Colombia

**DOI:** 10.1016/j.heliyon.2021.e08301

**Published:** 2021-11-02

**Authors:** Laura Navarro, Ricardo Camacho, Julián E. López, Juan F. Saldarriaga

**Affiliations:** a Department of Civil and Environmental Engineering, Universidad de los Andes, Carrera 1Este #19A-40, Bogotá, 111711, Colombia; b Department of Infrastructure Engineering, The University of Melbourne, Parkville, VIC, 3010, Australia; c Environmental Engineering Program, Universidad de Medellín, Carrera 87 #30-65, Medellín, 050026, Colombia

**Keywords:** Leaching, Partition coefficient, Imidacloprid, Lambda-cyhalothrin, Chlorpyrifos, Potential risk

## Abstract

Agricultural soils need monitoring systems to address pesticide risks for humans and the environment. The purpose of this paper was to obtain leaching risk maps of the pesticides imidacloprid, lambda-cyhalothrin, and chlorpyrifos in agricultural soil under an onion (*Allium cepa* L.) crop in Tibasosa, Boyacá, Colombia. This was obtained by studying the soil types in the area, analyzing the behavior of pollutants in the soil profile, using a delay factor and an attenuation factor to finally include GIS allowing visualization of the areas of greater potential risk in the study area.

## 1 Introduction

Sustainable agriculture depends to a large extent on healthy soils [1]. For this, more attention has gone towards monitoring pesticides in agricultural soils to determine potential environmental risks [2]. Pesticides have played an essential role in the green revolution by countering the attack of pests, which would otherwise reduce the quantity and quality of agricultural production, and have played an essential role in meeting the requirements of a rapidly growing population [3, 4]. Despite this, it has been proven that this green revolution has caused several problems such as loss of soil fertility, soil acidification, nitrate leaching, the resistance of species to pesticides, and loss of biological diversity [3, 5, 6].

The use of pesticides periodically deteriorates the situation, and repetitions for extended periods cause their accumulation in various environments due to their direct relationship, driving the ecosystem at risk due to its multiple toxicities [7]. The persistence of these chemicals in the environment is so frequent that their residues can remain in the soil and sediments for extensive periods after their supply to crops. After long periods of application times, the compounds could reach the water (surface and groundwater) through runoff and infiltration processes, reaching in some cases the food chain [3, 8].

Pesticides pollute both soil and water (surface and groundwater). Some enter the groundwater through runoff, dissolve in the water, or are adsorbed on the soil surface and eroded with the soil [9]. Pesticides also enter groundwater through drainage flow [10]. Leaching is the most common form of entry into groundwater and is washing water-soluble pesticides through the soil [11]. It is more likely to happen when the pesticide is soluble in water and does not adsorb on the soil's surface and sandy soil [10, 12]. Agricultural environmental frameworks aim to develop scientifically sound measures that can be used to assess the environmental risks associated with agricultural systems. As part of this assessment, pesticide leaching models are applied at large scales to assess the risk of groundwater contamination by these compounds in agricultural fields [13].

Models, indices, and indicators are used to determine the leaching potential of a chemical compound. Mainly models of potential groundwater contamination have been used based on the risk of pesticide leaching through the soil profile [14]. The model developed by Rao et al. [14] uses the attenuation factor (AF) and the delay factor (DF), which consider properties of soil pollutants (K_oc_ (organic carbon-water partitioning coefficient) and half-life), hydrological and climatic characteristics. The K_oc_ allows determining the mobility of pollutants and advection time. In this way, compounds with small K_oc_ values have shorter advection times to travel long distances in a short time and thus have an excellent leaching potential [15]. These indices allow determining the potential risk of pollutants leaching into groundwater bodies and establishing a discharge hazard classification. The importance of the delay factor and attenuation factor is that these indices consider the physicochemical characteristics of the pesticides and the characteristics of the soil.

In developing countries, most farmers use synthetic fungicides to manage foliar diseases, leading to contamination and loss of soil fertility, in addition to the environmental impacts associated with ecosystems. This is why, for the sustainable control of crop diseases, alternative strategies are needed to reduce the use of pesticides and the levels of environmental pollution [16]. The pesticide rate consumption depends on their agricultural area and the type of yield. Colombia is the third-largest consumer of pesticides (kg year^−1^) with 48, 618, 470, preceded by Italy and Turkey with 63,305,000 and 60,792,400, respectively. This large amount of land and the increase in the demand for products by the population makes the harvest efficiency higher. In addition, crop pests are a significant obstacle to productivity and profitability, considering that up to 45% of losses is due before and after harvest [17].

Worldwide, Colombia is the third country with the highest amount of land used for agriculture with 425,030 square kilometers, behind India (1,797,590), and Ecuador (749,770) [3,18]. Much of these lands have been used for many years for growing onions (*Allium cepa* Linn). Onions are grown in about 140 countries, using an estimated area of at least 5 million hectares worldwide [19]. Around 93 million tons of onion are produced worldwide [20]. The leading producer of this vegetable is China, with almost 23 million tons year^−1^; Colombia ranks 32 with an annual production of 484 thousand tons year^−1^. Onion belongs to the Amaryllidaceae family, and it is an important vegetable crop and one of the most used ingredients for condiments in sauces, stews, and soups [21]. Onion plantations are one of the crops with the highest demand for pesticides in their management practices. These polluting compounds pose a potential risk to human health and soil in onion crops [18]. Among the pests that most attack the onion crop is *Fusarium oxysporum*, classified as an important phytopathogen in this type of crop [22]. In the case of onion, WC Snyder & HN Hansen (FOC) has been identified, which causes basal rot of onions by *Fusarium* [19, 23].

Many field tests that use chemical fungicides have failed to reduce the symptoms associated with these pests. The aggressive use of fungicides can alter the soil microbiota, reducing beneficial soil microorganisms [19, 24]. Producers have not been able to harvest substantial portions of their onion crop due to *Fusarium* infection, and entire fields have been abandoned due to this pathogen. On the other hand, stored onions could be lost as symptoms may not be visible at harvest time [19, 25]. Chemical control becomes the only available and efficient solution to attack the problem. Within these are pesticides that have been an integral part of modern life and are used to protect soil from agriculture, grain storage, and others to eradicate pests that can transmit infectious diseases. These compounds are applied in many countries, and in developing countries such as Colombia, there is little technical support and regulation, making it easier for farmers to use them. Agricultural soils under onion plantations have a high level of pesticide contamination. However, tools for the environmental management of these contaminated soils in the country have not been developed. Colombia needs integral information and monitoring systems to address pesticide risks for humans and the environment.

For this reason, the main objective of this work is to determine the risk of leaching of various pesticides (imidacloprid, lambda-cyhalothrin, and chlorpyrifos). Furthermore, to analyze the behavior of pesticides in the soil profile using the delay factor and attenuation factor indices and include Geographic Information Systems (GIS) to visualize the areas of most significant potential risk in the selected study area.

## 2 Materials and methods

### 2.1 Study area and soils

**Figure 1:**
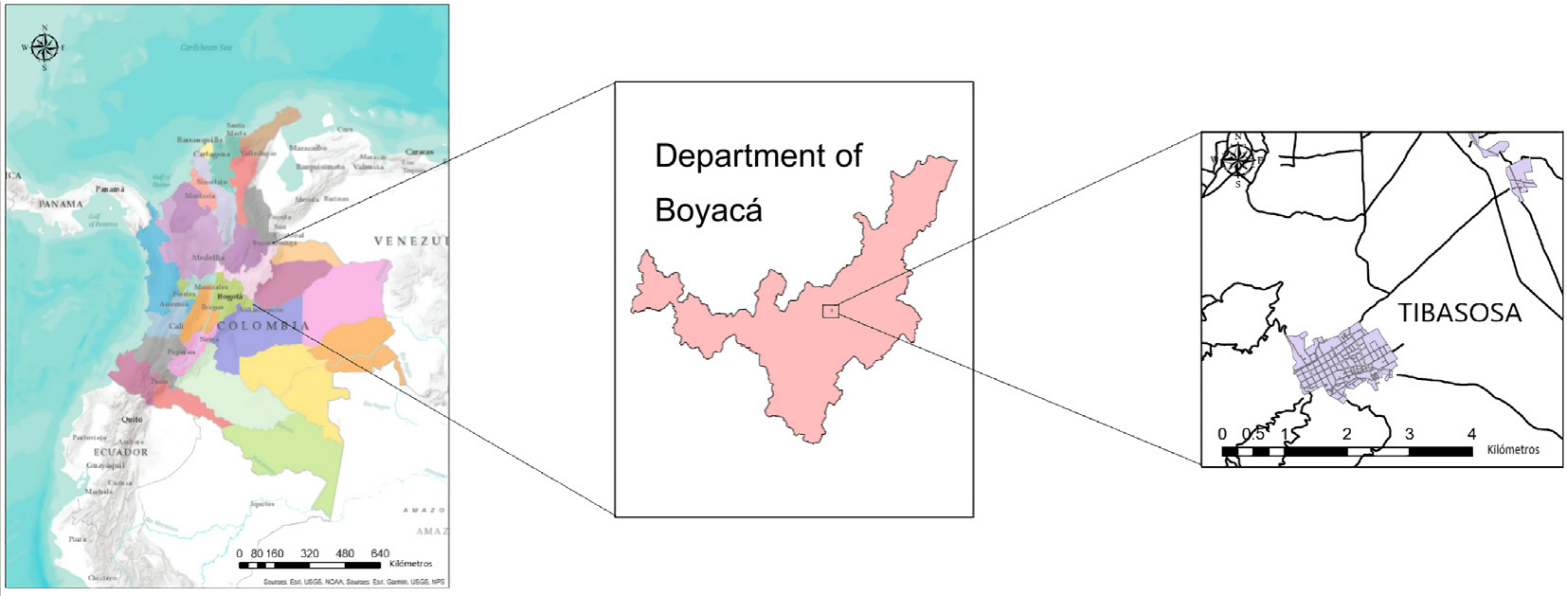
Study area location.

The municipality of Tibasosa is in the eastern Andes, in Boyacá, located in central-eastern Colombia (see Figure 1). It is part of the upper basin of the Chicamocha river; the zone covers an area of 1.44 km^2^ and is located at the coordinates 5.779276 N, -72.993673 E. This region has a humid climate with an average multi-year precipitation of 850.9 mm, an average temperature of 14 °C, and relative humidity in a range of 75 and 83%. The lithology of the area is predominantly sedimentary (see Figure 1).

The study area corresponds to an onion and potato crops area, to which aerial photographs and a Digital Terrain Model were obtained using an unmanned aerial vehicle (UAV). Information processing was carried out to analyze the soil types in the area based on the Semi-detailed Map of Acid Sulfated Soils of the Alto Chicamocha Irrigation District from 2014, scale 1: 25,000 [26].

The soils that make up this basin vary from soils with a low organic matter content to soils with high content. Four soil types have been found in the study area: Sulfic endoaquepts, Typic Sulfaquepts, Typic Sulfosaprist, and Typic Sulfohemists. A map has been constructed of the different horizons for each of the soil types, in which the different physicochemical characteristics present in Table S1 were specified. Each of the soil types was assigned a nomenclature in which limitation was specified by depth, sulfhydric materials, salinity, phreatic level, and pH, shown in Table 1.

**Table 1:** Definition of management units or phases according to the evaluation of characteristics and specific limitations in acid sulfate soils [27].

CLASSIFICATION OF LIMITATION BY DEPTH	LIMITING		
	Sulfuric Horizon ∗	Salinity ∗∗∗	Water table
	Hydrogen sulfide materials ∗∗	EC > 4 dS m^−1^	
	(a)	(s)	(n)
Very superficial (<20 cm)	a1	s1	n1
Superficial (≥ 20 < 50 cm)	a2	s2	n2
Moderately deep (≥ 50 < 80 cm)	a3	s3	n3
Deep (≥ 80 cm)	a4	s4	n4

∗pH < 3.5 and/or soluble sulfates <0.05% or evidence of speckles of jarosite or extractable sulfur >300 mg kg^−1^.

∗∗ pH > 4.0–6.0 and total sulfur >2% or extractable sulfur >800 mg kg^−1^; ∗∗∗ saline soil (dS m^−1^).

**Figure 2:**
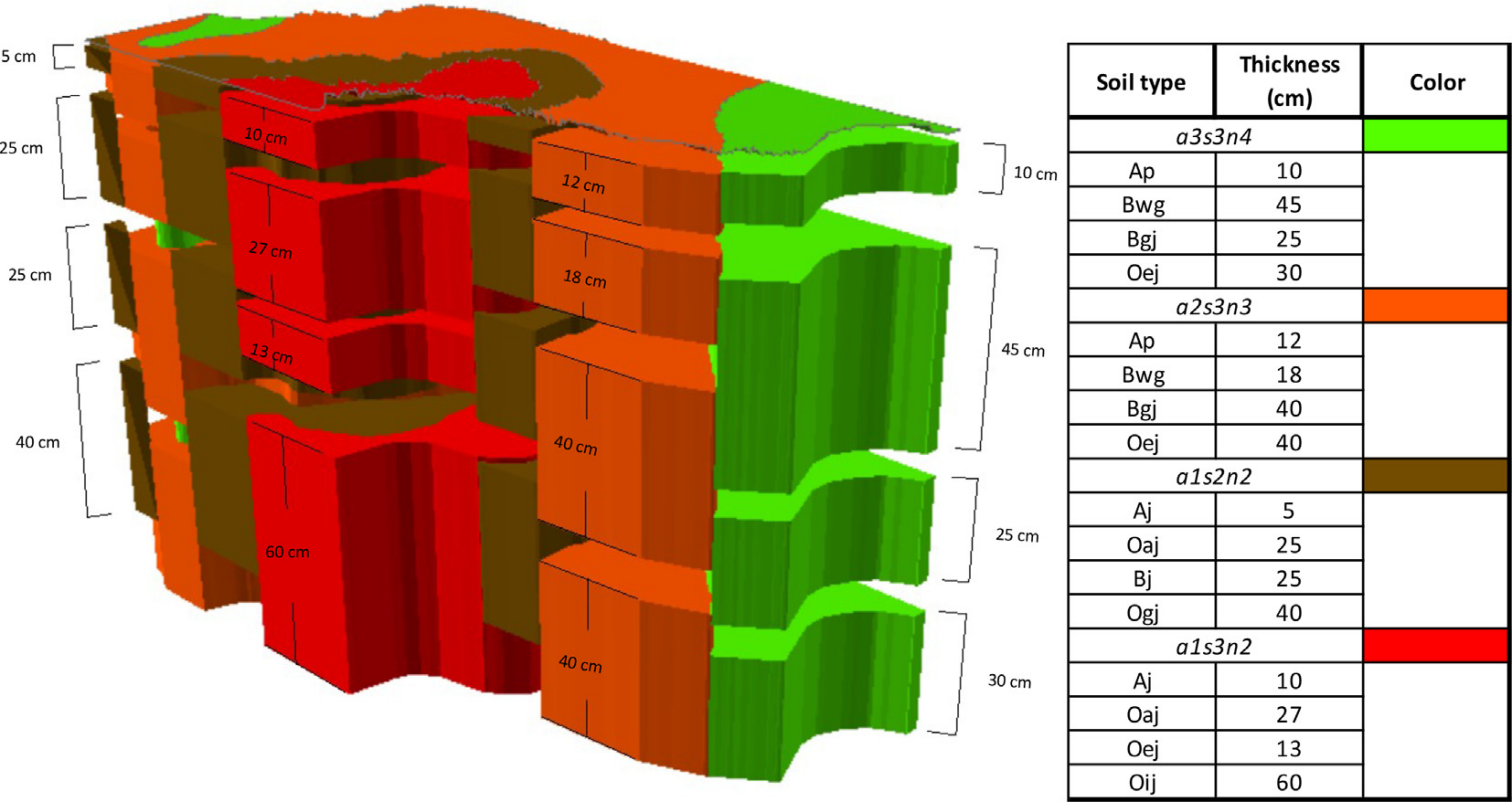
3D soil horizons map.

Additionally, to better visualize the soil profile for each of its types, a 3D map was constructed using the ESRI ArcScene 10.6.1 software, shown in Figure 2.

### 2.2 Crops

The crops that generally develop in this basin are wheat, potato, corn, barley, big-headed onion, among other permanent fruit crops [28]. Agro-industrial production is vital in this basin because Boyacá is considered the Department with the most significant onion production in Colombia. These crops are characterized by the intensive use of agrochemicals and irrigation, so they establish an exceptional environment for studying potential impacts on aquifers in the area.

### 2.3 Pesticides

For this work, three widely used pesticides have been determined in this area, imidacloprid, lambda-cyhalothrin, and chlorpyrifos, for which the K_oc_ value was obtained from the literature (Table 2). Likewise, half-life time values that represent the worst-case were determined. Generally, there are half-life ranges, since depending on the amount of organic matter that the soil contains, the pollutant can be easily degraded or not. For this reason, the worst case has been taken for which the contaminant has a very high half-life, which represents a significant risk of leaching [29, 30, 31].

**Table 2:** Physicochemical properties of pesticides.

Pesticide	K_oc_	Half-life time (days)	Soil degradation (days) (aerobic) DT50 (typical)	Solubility In water at 20 °C (mg L^−1^)	pH sensitivity	Reference
Imidacloprid	440	127	191 (persistent)	610	No	[32, 33]
Lambda-cyhalothrin	180000	30	175 (persistent)	0.05	No	[32, 33]
Chlorpyrifos	6070	30	386 (very persistent)	1.05	No	[32, 33]

### 2.4 Potential contamination index

Leaching potential has been determined for the three chosen pesticides, which are the most frequently used in the study area (Table 2).

The leaching potential has been determined according to the model proposed by Rao et al. [14], which establishes the determination of two indices: Attenuation Factor (AF) and Delay Factor (DF). The attenuation factor estimates the fraction of pesticide applied to the surface that leaches through the soil profile and is expressed with Eq. (1):

(1) $$AF=\exp\left[-\frac{0.693d\theta_{cc}DF}{qT_{\frac{1}{2}}}\right]$$

where *d* is the depth of the horizon in cm, *θ*_*cc*_ is the volumetric content of water in the soil at field capacity (m^3^ m^−3^), *q* is the net groundwater recharge in cm day^−1^, *T*_*1/2*_ is the life mean of herbicides in the soil (days).

The AFT coefficient that corresponds to the logarithmic transformation of AF has also been used for a better interpretation, which was carried out with the following formula (Eq. 2):

(2) $$AFT=\frac{\ln\;AF}{-0.693}$$

The DF coefficient indicates the ability of pesticides to leach through the soil, considering adsorption and behavior in the soil. This is defined by Eq. (3):

(3) $$DF=1+\frac{\rho_{d}focKoc}{\theta_{cc}}$$

where *ρ*_*d*_ is the bulk density of the soil (Mg m^−3^), *f*_*oc*_ is the organic carbon fraction (%), and *K*_*oc*_ is the organic carbon-water partitioning coefficient (L kg^−1^).

The calculations evaluated the mobility and behavior of the pesticides in each of the horizons according to the methodology proposed by Spadotto et al. [15] and Kookana et al. [34]. With these indices, pesticides were classified depending on mobility and leaching potential using the classification proposed by Khan and Liang [35] presented in Table 3. Table 3 shows the classification from very likely to very unlikely infiltration and the arrival of the pesticide to the aquifer. This classification is given according to the found ranges of AF and AFT.

**Table 3:** Pesticide classification [36].

DF	Classification	AF	AFT	Classification
= 1	very mobile	≥2.5 × 10^−1^ y ≤ 1	≤2	very likely
>1 y < 2	mobile	≥1 × 10^−1^ y < 2.5 × 10^−1^	≥2 y < 3	likely
≥2 y < 3	moderately mobile	≥1 × 10^−2^ y < 1 × 10^−1^	≥3.3 y < 7.2	moderately likely
≥3 y < 10	moderately immobile	≥1 × 10^−4^ y < 1 × 10^−2^	≥7.2 y < 13.3	unlikely
≥10	very immobile	<1 × 10^−4^	>13.3	very unlikely

### 2.5 Net groundwater recharge

To calculate the AF and DF coefficients, it has been needed to obtain the value of the net groundwater recharge. The net groundwater recharge refers to the annual amount of water that penetrates the soil, expressed in mm/year. This is an essential factor in quantifying the potential for contamination of underground aquifers because it can facilitate the transport of pollutants through the soil profile.

**Figure 3:**
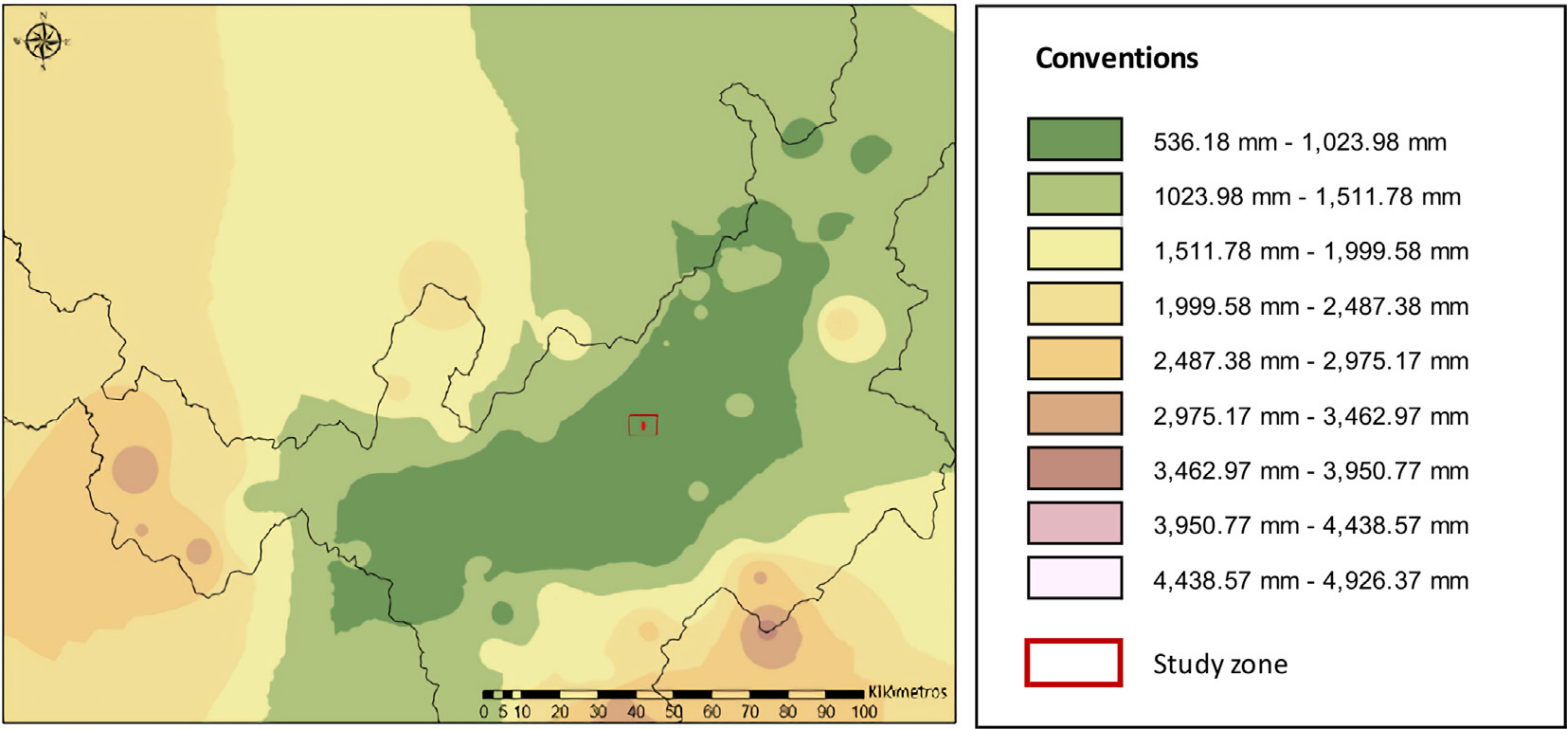
Precipitation map for the Department of Boyacá.

The value used is obtained from a general water balance between precipitation and evapotranspiration. Using information from the IDEAM weather stations in the Boyacá region from 1981 to 2010, an interpolation has been carried out by the Inverse Distance Weighting or IDW method, which assigns weights to the environment data an inverse function of the distance that the to stop. With this, a precipitation map was obtained for the region (Figure 3).

As shown in Figure 3, the study area has an annual rainfall between 533 mm and 4926 mm per year, with temperatures between 23 °C and 28 °C. In addition, sandy soils correspond to an area with recharge values greater than 600 mm per year; it is an area of high-water recharge [37].

### 2.6 Risk maps

The DF and AFT indices were calculated, and the information presented in risk maps, developed in ESRI ArcMap 10.6, for each chosen pesticide.

### 2.7 Data analysis

A multivariate analysis was used to investigate possible correlations between pesticide mobility and soil organic matter content.

## 3 Results and discussion

This study shows that the application of leaching models is a valuable tool for the environmental management of agricultural soils. Various relevant findings were identified: (i) the mobility and risk of leaching was dependent on the physicochemical properties of the soil horizons, (ii) imidacloprid would be a target contaminant in soil management under onion plantations in Colombia, and (iii) risk maps are helpful to observe the behavior of pesticide leaching in soil profiles. The implications of these findings are herein discussed to contribute to the environmental management of pesticide-contaminated soils in Colombia and other developing countries sharing similar soil and climatic conditions.

**Figure 4:**
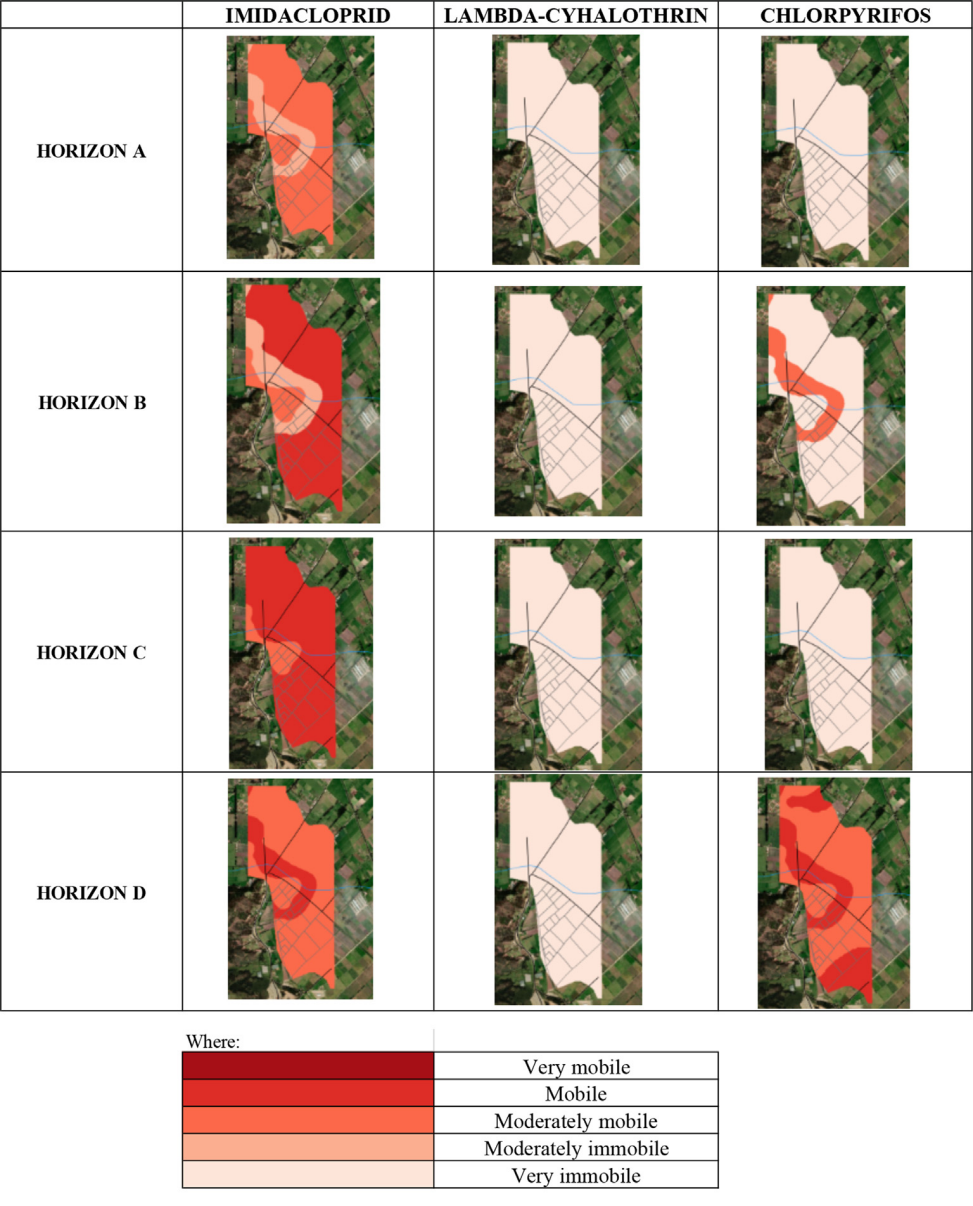
DF risk maps according to pesticides and horizons.

In Figure 2, the study area was divided into four soil horizons, which for this work have been called horizon A, horizon B, horizon C, and horizon D. In Table S2, we present the results for each of the pesticides along with the soil profile. Figure 4 shows the risk maps obtained for each of the pesticides studied and each horizon.

According to the properties shown in Table 2, it can be seen that all the pesticides studied have a high average lifetime and are classified as persistent or very persistent (recalcitrant). Hence, their biodegradation in the soil is limited [38, 39, 40]. Furthermore, in the case of chlorpyrifos and lambda-cyhalothrin, the low solubility in water increases their persistence in soils. Together with the models in Figure 4, these data show the high risk that these pesticides present in the soil and subsoil of the study area. Another big problem with these pesticides is that they are not sensitive to changes in pH, which indicates that chemical degradation, for example, alkaline hydrolysis is unlikely. This makes their residence in the soil much more significant and the occurrence of negative impacts on surface and groundwater.

The mobility of pesticides in soils is subject to physical, chemical, and biological processes, so these compounds can be transferred to lower horizons or remain in the soil surface layers; both can generate long-term damage to ecosystems and the health of the inhabitants. The models proposed in the study suggest greater mobility of imidacloprid and chlorpyrifos in the soil, which is high throughout the entire profile for imidacloprid. In contrast, chlorpyrifos is high in horizon A and decreases as it descends in the ground profile. The mobility characteristics found and high half-life times make this type of pesticide in the soil quite problematic. In the short and medium term, they cause problems to public health through exposure routes such as ingestion or dermal [41]. Because these compounds are highly toxic and persistent in the environment, the results indicate a significant environmental risk from pesticides in the study area. Likewise, resistance to pests and diseases could be generated in crops due to the resistance they can acquire to these pesticides [42].

In Colombia, other studies have reported the presence and mobility of some persistent organic compounds such as 4,4′-DDT, 4,4-DDD, alpha-chlordane, and lindane used in agronomic practices of crop management. These compounds were found mainly distributed in the first 30 cm of the soil profile, despite not being applied for 20 years in the area, confirming their persistence in the environment [24, 43, 44, 45, 46].

**Figure 5:**
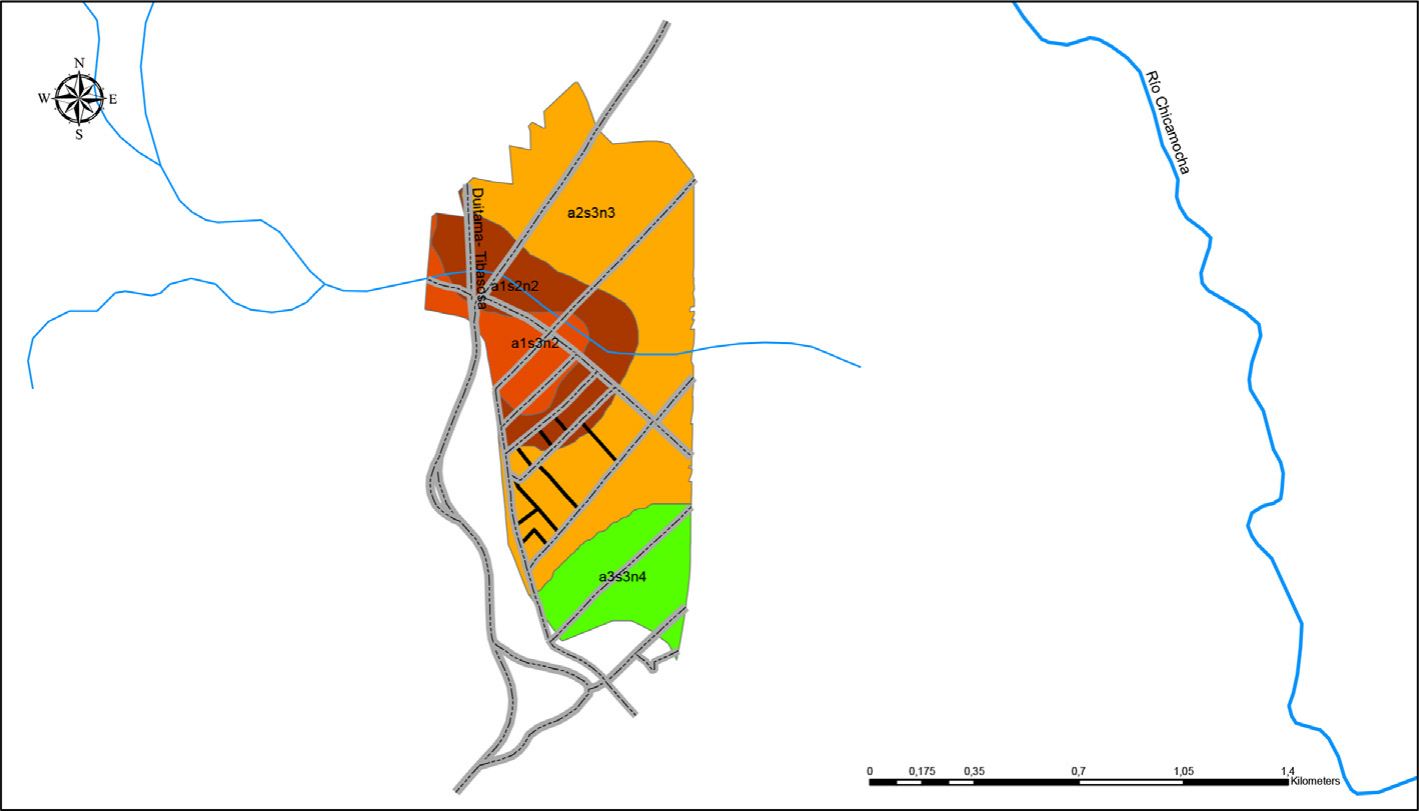
Map of soil types of the study area.

Considering the types of soil presented in Figure 5, it has been observed greater mobility for the pesticide imidacloprid in all horizons and a high probability of leaching. In contrast, the pesticide lambda-cyhalothrin has low mobility and a low probability of leaching in all its horizons. Finally, there is little mobility in the first horizon for the pesticide chlorpyrifos and a reasonable probability of leaching. However, there are zones of moderate mobility and high mobility in the last horizon, which is combined with its probability of leaching for these same zones. The previous indicates that the three pesticides under study have a moderate to high risk of leaching into the aquifer and even reaching groundwater, leading to potential contamination of the ecosystem, mainly affecting the fauna and flora of the area.

**Figure 6:**
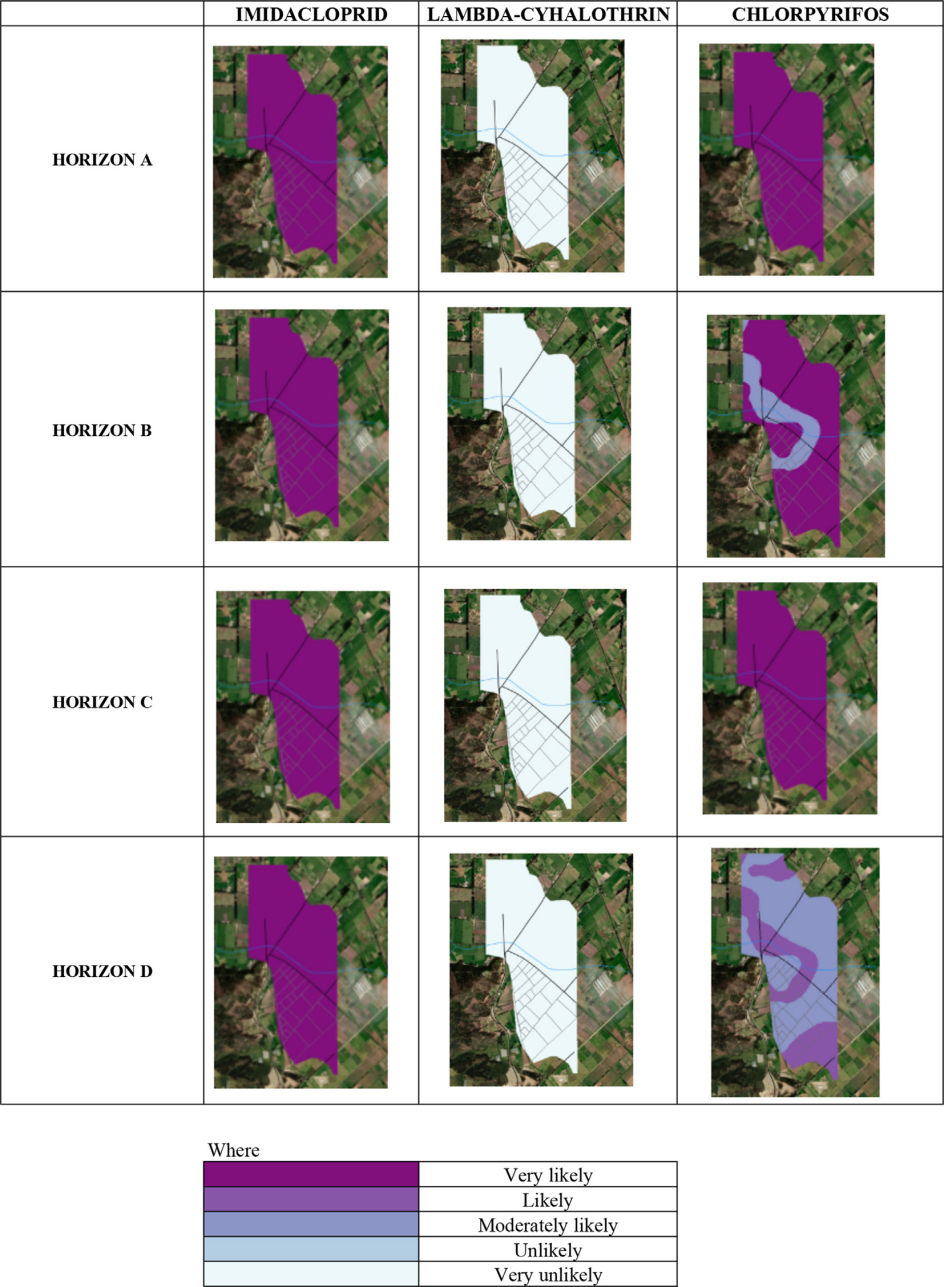
AF risk maps according to pesticides and horizons.

This risk is associated with the physicochemical properties of the three pesticides, which have a high resistance to degradation in the soil (Table 2). These pesticides are also classified as persistent and, in the case of chlorpyrifos, as very persistent. Figure 6 presents the high risk of these pesticides, especially for imidacloprid and chlorpyrifos, which implies a high impact on the soils flora and fauna. Crop pests are being attacked with these and affecting the native flora and fauna of the study area. Similarly, according to the persistence of these compounds (Table 2) and the results of the AF risk map (Figure 4), it should be considered in the soil remediation plans, in the cultivation process and the environmental impacts due to tillage the contamination of groundwater and surface waters that these three compounds can cause in the area. This is because there is a strong probability of leaching, especially lambda-cyhalothrin, which is shown to be the one with the highest mobility with respect to the other two pesticides.

**Figure 7:**
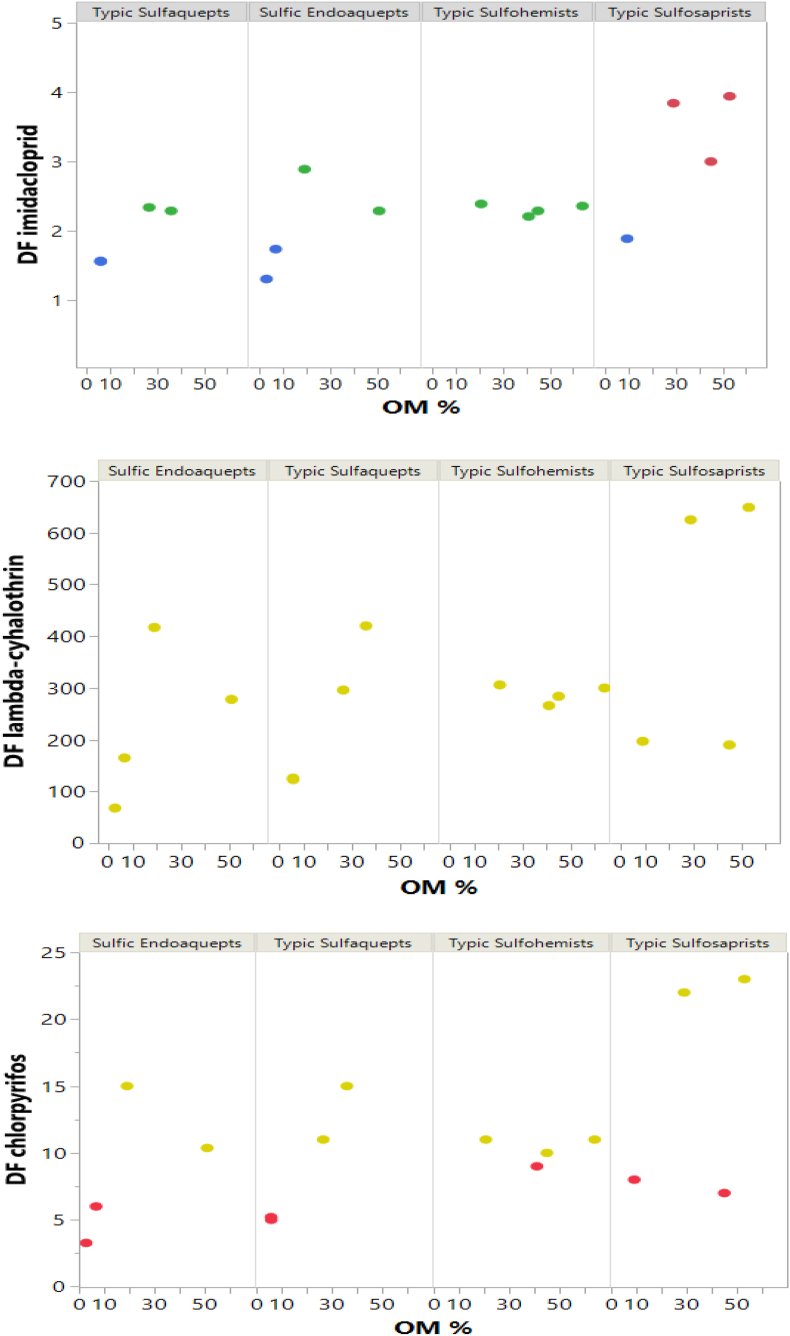
Relationship between organic matter and DF for each pesticide according to soil type. Blue: Mobile. Green: Moderately mobile. Red: Moderately immobile. Yellow: Very immobile.

**Figure 8:**
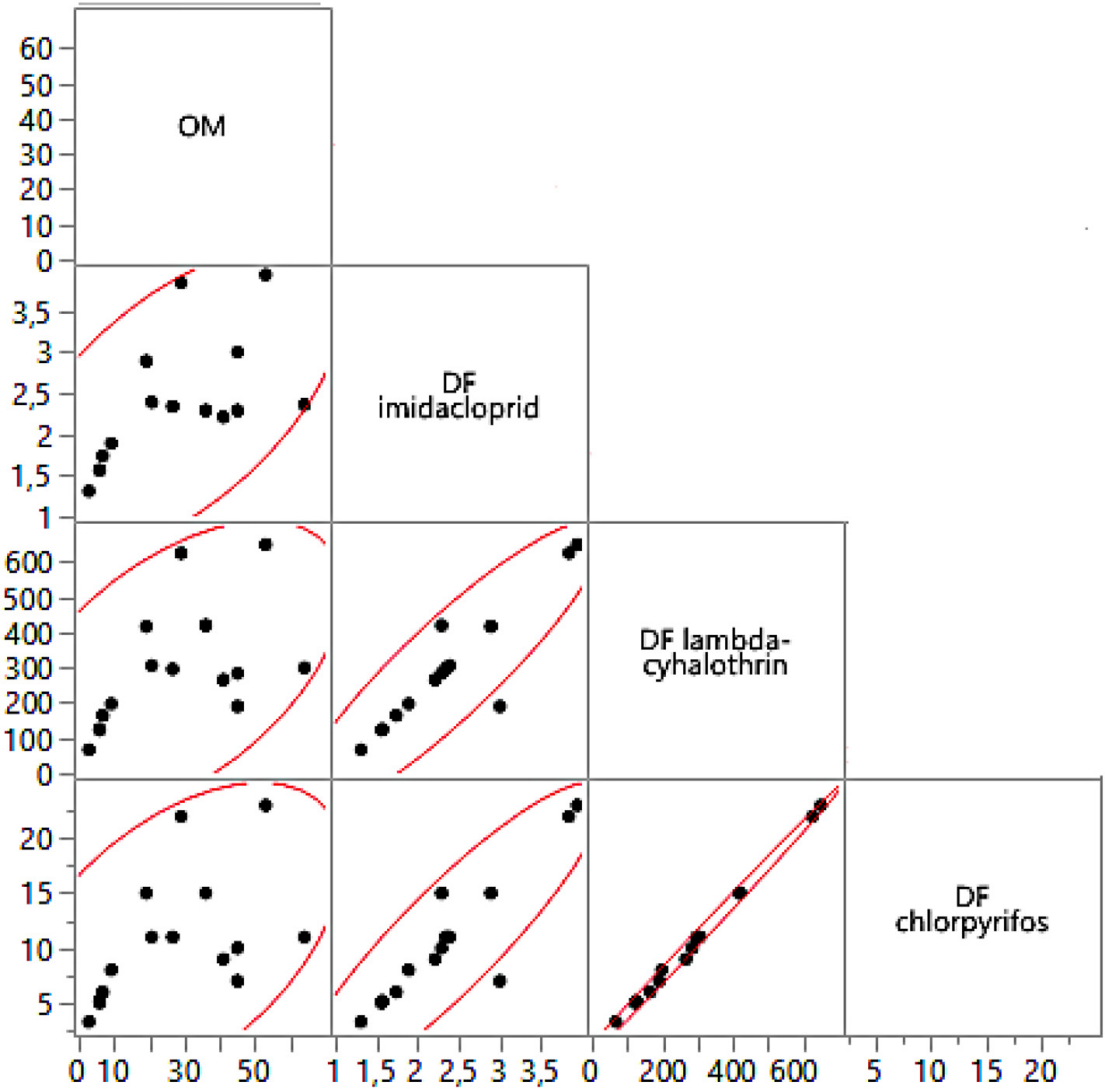
Pearson correlation analysis between DF values obtained for pesticides and soil organic matter content.

The behavior of the potential risk of leaching in the studied area is directly related to the physicochemical properties of the soil, and it is affected as the depth increases relative to the surface. Characteristics such as the amount of organic matter and bulk density impact the behavior of pesticides in the soil due to processes such as degradation, adsorption, and desorption [36]. Especially, soil organic matter content has a significant impact on the fate of organic contaminants in soils due to the high sorption capability of this colloid and favoring the microbial degradation of pesticides [47]. As shown in Figure 7, increasing the organic matter content in soils reduces the mobility of pesticides, regardless of soil type. Pearson correlation analysis (Figure 8) indicates a significant positive correlation (P < 0.05) between DF values of each pesticide and organic matter content (r = 0.62 to 0.71). Therefore, as soil organic matter increases, the mobility of pesticides decreases. According to the results, soils could retain more pesticides in the horizons with higher organic matter content (e.g., horizons O and A). However, the risk associated with this type of contaminants will depend not only on their mobility, but also on the biochemical changes associated with microbial activity [47], which should be studied.

Another critical factor in evaluating the risk maps of pesticides and their impact on the environment is the half-life values. In the case of imidacloprid, we obtained the highest value, which directly relates to its high potential risk of leaching due to its degradation rate in the soil compared to the other two pesticides. Similarly, when comparing the *K*_*oc*_ values, we observed that the lowest constitutes imidacloprid. According to Spadotto et al. [15], this compound can travel distances in a shorter time than other pesticides, thus increasing its leaching potential. Likewise, it has also been proven that soils with a high content of organic matter or the addition of mature compost can provide a high potential for retention of this type of pesticide in the soil, resulting in a high potential for remobilization and toxicity to plants [48].

On the other hand, lambda-cyhalothrin is known to dissipate rapidly from water; only 30% has been found to remain in the aqueous phase [49]. This characteristic aligns with our results of it having the most negligible probability of permanence in the soil matrix; its physicochemical properties allow its dissipation in the water and prevent its leaching into the soil.

In the case of chlorpyrifos, it is moderately persistent in temperate soils, and the half-life depends on the type of soil and environmental conditions [50, 51, 52]. The results regarding chlorpyrifos are consistent with those found in other studies saying that the probability of transporting this pesticide is low or moderately low. It makes the risk of contamination of the aquifer in the study area lower than imidacloprid, which turns out to be the one with the highest mobility among the three pesticides studied.

In this way, it is possible to observe that the half-life time and the partition coefficient of organic carbon have an inversely proportional relationship to determine the potential risk of leaching a contaminant. It is essential to consider using specific data (half-life time and partition coefficient of organic carbon) of the zone in further studies. However, under the models applied in this study, the use of imidacloprid presents a greater risk of contamination.

## 4 Conclusions

The modeling studies and risk map analysis of these three pesticides have helped to elucidate the behavior that these may have in the soil, which will serve in the future to develop environmental remediation plans and strategies in the zone. Lambda-cyhalothrin and chlorpyrifos have been shown to have lower risks of movement, thus reducing their risk of affecting groundwater and surface water. However, imidacloprid does present a greater risk of contamination of the aquifers and mobility, becoming a potential pollutant. According to this study, the use of these pesticides in the area should be avoided, or even after applying them and harvesting, carry out bioremediation processes. The maps obtained are essential because they serve to carry out soil and water remediation plans. Observing the movement and persistence of these compounds in the soil allows one to search for alternatives such as phytoremediation or bioremediation, considering the mobility in the matrix. Likewise, these risk maps have made it possible to observe the behavior of the pesticides leaching in the soil profile. It has been possible to determine the transport dynamics. Therefore, the affectation order of the aquifers in the study area in Tibasosa, Colombia, from highest to lowest risk is imidacloprid > chlorpyrifos > lambda-cyhalothrin.

It is necessary to reduce this type of pesticide in the agricultural practices of onion cultivation because there is a high risk of persistence, infiltration, and contamination of the three compounds studied, with a more significant impact of the imidacloprid. We recommend avoiding using the latter since properties in the soil allow its infiltration and runoff, causing a tremendous environmental problem. The other two compounds can remain for long periods. In processes of soil tillage and crop rotation, typical of the area, these could be leached by runoff or infiltrate and cause major environmental problems in the area or downstream of it.

## Declarations

### Author contribution statement

Laura Navarro: Performed the experiment; Analyzed and interpreted the data.

Ricardo Camacho: Conceived and designed the experiments; Analyzed and interpreted the data; Contribute reagents, materials, analysis tools; Wrote the paper.

Julián E. López: Performed the experiment; Analyzed and interpreted the data; Wrote the paper.

Juan F. Saldarriaga: Conceived and designed the experiments; Performed the experiment; Analyzed and interpreted the data; Contribute reagents, materials, analysis tools; Wrote the paper.

### Funding statement

This work was supported by Department of Civil and Environmental Engineering at 10.13039/501100006070Universidad de los Andes. This publication was partially made possible by call for proposals CI-0120: "Publish your new knowledge or expose new creations" from the Office Vice President for Research and Creation at Universidad de los Andes.

### Data availability statement

Data included in article/supplementary material/referenced in article.

### Declaration of interests statement

The authors declare no conflict of interest.

### Additional information

No additional information is available for this paper.
