# Supplementary material for: Assessment of the potential risk of leaching pesticides in agricultural soils: study case Tibasosa, Boyacá, Colombia
Source: Heliyon. 2021 Nov 2;7(11):e08301. doi: 10.1016/j.heliyon.2021.e08301 (PMC8591474; doi:10.1016/j.heliyon.2021.e08301)
Supplement: supplementary_materials [file mmc1.docx]

Assessment of the potential risk of leaching pesticides in agricultural soils: study case Tibasosa, Boyacá, Colombia

Laura Navarro^1^, Ricardo Camacho^1,2^, Julián E. López^3^, Juan F. Saldarriaga^1,*^

^1^ Department of Civil and Environmental Engineering, Universidad de los Andes, Carrera 1Este #19A-40, Bogotá, Colombia, 111711

^2^ Department of Infrastructure Engineering, The University of Melbourne, Parkville, Australia, VIC 3010

^3^ Environmental Engineering Program, Universidad de Medellín, Carrera 87 #30-65, Medellín, Colombia, 050026

* Correspondence: jf.saldarriaga@uniandes.edu.co

**Supplementary materials**

**Table S1:** Physicochemical properties of the soil in the study area

|  |  | **OM** | **pH** | **EC** | **Al** | **H** | **Ca** | **Mg** | **K** | **Na** | **CICE** | **P** | **Mn** | **Fe** | **Zn** | **Cu** | **B** | **S** |
| --- | --- | --- | --- | --- | --- | --- | --- | --- | --- | --- | --- | --- | --- | --- | --- | --- | --- | --- |
| Modal profile | Horizons | % |  | dS m^-1^ | Mg kg^-1^ | | | | | | Cmol (+) kg^-1^ | Mg kg^-1^ | | | | | | |
| PSMI-02 | Ap 0-10 | 19.12 | 5.36 | 1.6 | 0.05 | 0.18 | 23.6 | 2.96 | 1.86 | 0.56 | 29.23 | 11.9 | 5.53 | 556 | 2.5 | 0.93 | 1 | 76.8 |
| Sulfic Endoaquepts | Bwg 10-55 | 2.85 | 4.23 | 0.65 | 5.4 | 0.5 | 5.07 | 0.97 | 0.51 | 0.25 | 12.7 | 7.3 | 3.7 | 133 | 2.25 | 0.69 | 0.28 | 161 |
|  | Bgj 55- 80 | 6.79 | 3.69 | 3.5 | 6.2 | 0.4 | 8.95 | 0.96 | 0.28 | 0.01 | 16.8 | 7.7 | 5.93 | 247 | 1.77 | 0.4 | 0.51 | 308 |
|  | Oeg 80 -110 | 51 | 4.3 | 4.1 | 4 | 0.8 | 10.8 | 3.24 | 1.35 | 0.01 | 20.15 | 59.6 | 6.92 | 531 | 1.48 | 0.27 | 1.3 | 1715 |
| PSDU-01 | Ap 0-12 | 16.6 | 3.92 | 0.38 | 12.1 | 1 | 0.96 | 0.35 | 0.61 | 0.01 | 15.03 | 18 | 2.01 | 380 | 0.97 | 0.21 | 0.36 | 168 |
| Typic Sulfaquepts | Bwg 12-30 | 5.9 | 3.81 | 0.56 | 12.3 | 0.1 | 1.43 | 0.21 | 0.32 | 0.05 | 14.41 | 13.1 | 1.19 | 98.7 | 0.57 | 0.21 | 0.25 | 286 |
|  | Bgj 30-70 | 5.85 | 3.5 | 2.66 | 9.7 | 0.8 | 6.4 | 0.47 | 0.14 | 0.01 | 17.52 | 18.6 | 4.92 | 214 | 0.51 | 0.18 | 0.17 | 951.2 |
|  | Oej 70-110 | 36 | 3.5 | 4.35 | 18.8 | 0.8 | 9.26 | 2.79 | 0.46 | 1.1 | 33.21 | 57.4 | 26.8 | 223 | 0.99 | 0.42 | 0.6 | 1563 |
| PSVU-03 | Aj 10-15 | 29 | 3.18 | 2.1 | 11.3 | 2 | 2.1 | 0.91 | 0.34 | 0.46 | 17.11 | 18.1 | 6.79 | 386 | 0.52 | 0.1 | 0.93 | 2609 |
| Typic Sulfosaprists | Oaj 15-35 | 53 | 3 | 3.4 | 12.4 | 0.05 | 2.38 | 1.17 | 0.14 | 1.6 | 17.69 | 12.3 | 10.6 | 224 | 0.59 | 0.08 | 0.83 | 621 |
|  | Bj 35-60 | 9.18 | 3.34 | 3.4 | 13.2 | 1.2 | 6.8 | 3.37 | 0.47 | 3.83 | 28.87 | 37.6 | 21.8 | 456 | 0.57 | 0.32 | 1.1 | 829 |
|  | Ogj 60-100 | 45 | 6.5 | 8.1 | 5.5 | 3.7 | 4.7 | 5.52 | 0.84 | 1.2 | 21.46 | 181 | 28.9 | 131 | 0.94 | 0.24 | 1 | 2477 |
| PSVA-02 | Aj 0-10 | 20.7 | 3.3 | 1.3 | 10.9 | 1 | 0.7 | 0.09 | 0.21 | 0.01 | 12.91 | 13 | 1.59 | 259 | 1.09 | 0.17 | 0.63 | 791 |
| Typic Sulfohemists | Oaj 10-37 | 41 | 3.19 | 1.3 | 12.2 | 8.1 | 0.91 | 0.13 | 0.18 | 0.01 | 21.53 | 6.2 | 2.73 | 348 | 0.78 | 0.1 | 0.57 | 941 |
|  | Oej 37-50 | 45 | 3.35 | 2.1 | 23.5 | 0.3 | 1.23 | 0.24 | 0.2 | 0.11 | 25.58 | 18.5 | 12.5 | 197 | 4.79 | 0.09 | 0.76 | 1298 |
|  | Oij 50-110 | 64 | 3.16 | 4.9 | 23 | 10 | 2.6 | 0.4 | 0.36 | 0.01 | 36.37 | 10.5 | 7.6 | 140 | 3.1 | 0.1 | 0.62 | 1365 |

OM: Organic matter

CICE: Effective cation exchange capacity

EC: Electrical conductivity

|  |  | **Al** | **Ca** | **Mg** | **K** | **Na** | **Pw** | **Ar** | **L** | **A** | **Text** | **ρ_d_** | **ρ_r_** | **Pt** |
| --- | --- | --- | --- | --- | --- | --- | --- | --- | --- | --- | --- | --- | --- | --- |
| Modal profile | Horizons | % | | | | | % | | | |  | g cm^-3^ | g cm^-3^ | % |
| PSMI-02 | Ap 0-10 | 0.17 | 80.81 | 10.13 | 6.36 | 1.92 | 34.6 | 32.7 | 16.4 | 51 | FArA | 1.2 | 2.07 | 51.2 |
| Sulfic Endoaquepts | Bwg 10-55 | 42.52 | 39.92 | 7.64 | 4.02 | 1.97 | 44.2 | 66.3 | 32 | 1.7 | Ar | 1.3 | 2.39 | 49.9 |
|  | Bgj 55- 80 | 36.9 | 53.27 | 5.71 | 1.67 | 0.06 | 51.3 | 55.6 | 42.7 | 1.7 | Arl | 1.33 | 2.5 | 50.8 |
|  | Oeg 80 -110 | 19.85 | 53.35 | 16.08 | 6.7 | 0.05 | 89.7 |  |  |  |  | 0.3 | 1.74 | 52.6 |
| PSDU-01 | Ap 0-12 | 80.51 | 6.39 | 2.33 | 4.06 | 0.07 | 86.3 | 76.9 | 22.1 | 1 | Ar | 1.07 | 2.28 | 53.2 |
| TYPIC SULFAQUEPTS | Bwg 12-30 | 85.36 | 9.92 | 1.46 | 2.22 | 0.35 | 54.3 | 68.6 | 30.1 | 1.3 | Ar | 1.25 | 2.39 | 47.8 |
|  | Bgj 30-70 | 55.37 | 36.53 | 2.68 | 0.8 | 0.06 | 60.5 | 77.3 | 22.5 | 0.2 | Ar | 1.29 | 2.45 | 47.5 |
|  | Oej 70-110 | 56.61 | 27.88 | 8.4 | 1.39 | 3.31 | 74.3 | 42.4 | 17 | 40.6 | Ar | 0.7 | 2.18 | 57.8 |
| PSVU-03 | Aj 10-15 | 66.04 | 12.27 | 5.32 | 1.99 | 2.69 | 42.5 | 65.5 | 10.9 | 1.8 | Ar | 1.18 | 2.28 | 48.4 |
| TYPIC SULFOSAPRISTS | Oaj 15-35 | 69.81 | 13.45 | 6.61 | 0.79 | 9.04 | 82.2 | 43.1 | 21.5 | 35.4 | Ar | 0.67 | 2.03 | 67 |
|  | Bj 35-60 | 45.72 | 23.55 | 11.67 | 1.63 | 13.27 | 82.4 | 76.5 | 7.52 | 0.04 | Ar | 1.17 | 2.11 | 44.5 |
|  | Ogj 60-100 | 25.63 | 21.9 | 25.72 | 3.91 | 5.59 | 318.7 | - |  |  |  | 0.23 | 2.03 | 88.6 |
| PSVA-02 | Aj 0-10 | 84.43 | 5.42 | 0.7 | 1.63 | 0.08 | 65 | 67.2 | 25.3 | 7.5 | Ar | 0.82 | 2.15 | 61.9 |
| TYPIC SULFOHEMISTS | Oaj 10-37 | 56.67 | 4.23 | 0.6 | 0.84 | 0.05 | 175.1 | 20.2 | 13.5 | 66.3 | FArA | 0.36 | 1.6 | 78.2 |
|  | Oej 37-50 | 91.87 | 4.81 | 0.94 | 0.78 | 0.43 | 204 | - | 20.4 | 79.7 | FA | 0.35 | 1.93 | 81.9 |
|  | Oij 50-110 | 63.24 | 7.15 | 1.1 | 0.99 | 0.03 | 282.8 | - |  |  |  | 0.26 | 1.83 | 85.9 |

Pw: water content for a given irrigation threshold

Ar: clay

L: lime

A: sand

Text: texture

ρ_d_: bulk density

ρ_r_: real density

Pt: percentage of total pores

|  | **Pore distribution** | | | **Moisture** | | | **Dry** | | | **Consistency (%H)** | | |
| --- | --- | --- | --- | --- | --- | --- | --- | --- | --- | --- | --- | --- |
| Modal profile | Macropore  % | Meso and  micropore  % | CD | DGM | DPM | EA (%) | DGM | DPM | EA (%) | LL | LP | IP |
| PSMI-02 | 17.31 | 33.88 | 1.11 | 1.39 | 2.97 | 65.3 | 3.18 | 4.2 | 91.8 | 82 | 57 | 25 |
| Sulfic Endoaquepts | 21.21 | 28.65 | 28.65 | 2.8 | 4.57 | 82.8 | 5.2 | 5.76 | 98.1 | 58 | 28 | 30 |
|  |  |  |  |  |  |  |  |  |  |  |  |  |
|  |  |  |  |  |  |  |  |  |  |  |  |  |
| PSDU-01 | 27.33 | 25.83 | 0.22 | 1.45 | 3.14 | 65.2 | 4.3 | 5.02 | 96.6 | 96 | 73 | 23 |
| Typic Sulfaquepts | 11.52 | 36.25 | 0.26 | 2.67 | 5.04 | 74.8 | 6.51 | 6.71 | 99.4 | 70 | 39 | 31 |
|  |  |  |  |  |  |  |  |  |  |  |  |  |
|  |  |  |  |  |  |  |  |  |  |  |  |  |
| PSVU-03 | 14.68 | 33.67 | 0.28 | 0.79 | 2.27 | 46.2 | 4.14 | 4.97 | 96.5 | 66 | 41 | 25 |
| Typic Sulfosaprists | 14.71 | 52.29 | 0.68 | 0.73 | 1.82 | 47.6 | 3.91 | 4.81 | 94.2 | 135 | 90 | 45 |
|  |  |  |  |  |  |  |  |  |  |  |  |  |
|  |  |  |  |  |  |  |  |  |  |  |  |  |
| PSVA-02 | 16.15 | 45.71 | 0.37 | 1.04 | 2.34 | 59.7 | 3.3 | 4.15 | 94.4 | 104 | 74 | 30 |
| Typic Sulfohemists | 20.24 | 57.93 | 0.79 | 0.94 | 2.89 | 45 | 6.07 | 6.39 | 99.1 | 212 | 106 | 106 |
|  |  |  |  |  |  |  |  |  |  |  |  |  |
|  |  |  |  |  |  |  |  |  |  |  |  |  |

DGM: mean geometric diameter

DPM: average weighted diameter

EA: State of aggregation

LL: liquid limit

LP: plastic limit

IP: plasticity index

**Table S2:** Evaluation of the behavior of pesticides in the soil profile of the study area

**Imidacloprid**

| **Soil type** | **ρ_d_** | **f_oc_ %** | **d** | **ϕ_cc_ %hg** | **DF** | **AF** | **AFT** | **INT DF** | **Classification** |
| --- | --- | --- | --- | --- | --- | --- | --- | --- | --- |
| a3s3n4_Ap | 1.2 | 0.1110 | 10 | 57.61 | 2.89 | 9.66.E-01 | 0.0503 | Moderately mobile | Very likely |
| a2s3n3_Ap | 1.07 | 0.0965 | 12 | 62.94 | 2.34 | 9.64.E-01 | 0.0534 | Moderately mobile | Very likely |
| a1s2n2_Aj | 1.18 | 0.1686 | 10 | 57.31 | 3.84 | 9.55.E-01 | 0.0664 | Moderately immobile | Very likely |
| a1s3n2_Aj | 0.82 | 0.1203 | 10 | 58.14 | 2.39 | 9.71.E-01 | 0.0419 | Moderately mobile | Very likely |
| a3s3n4_Bwg | 1.3 | 0.0166 | 45 | 57.61 | 1.31 | 9.32.E-01 | 0.1023 | Mobile | Very likely |
| a2s3n3_Bwg | 1.25 | 0.0343 | 18 | 62.94 | 1.56 | 9.64.E-01 | 0.0533 | Mobile | Very likely |
| a1s2n2_Oaj | 0.67 | 0.3081 | 25 | 57.31 | 3.94 | 8.88.E-01 | 0.1707 | Moderately immobile | Very likely |
| a1s3n2_Oaj | 0.36 | 0.2384 | 27 | 58.14 | 2.21 | 9.30.E-01 | 0.1046 | Moderately mobile | Very likely |
| a3s3n4_Bgj | 1.33 | 0.0395 | 25 | 57.61 | 1.74 | 9.49.E-01 | 0.0759 | Mobile | Very likely |
| a2s3n3_Bgj | 1.29 | 0.0340 | 40 | 62.94 | 1.57 | 9.21.E-01 | 0.1194 | Mobile | Very likely |
| a1s2n2_Bj | 1.17 | 0.0534 | 25 | 57.31 | 1.89 | 9.45.E-01 | 0.0818 | Mobile | Very likely |
| a1s3n2_Oej | 0.35 | 0.2616 | 13 | 58.14 | 2.29 | 9.64.E-01 | 0.0522 | Moderately mobile | Very likely |
| oa2s3n3_Oej | 0.7 | 0.2093 | 40 | 62.94 | 2.90 | 8.58.E-01 | 0.2208 | Moderately mobile | Very likely |
| a1s2n2_Ogj | 0.23 | 0.2616 | 40 | 57.31 | 1.86 | 9.15.E-01 | 0.1287 | Mobile | Very likely |
| a1s3n2_Oij | 0.26 | 0.3721 | 60 | 58.14 | 2.36 | 8.42.E-01 | 0.2487 | Moderately mobile | Very likely |

**ρ_d_: bulk density of dry soil**

**f_oc_: organic carbon fraction**

**d: depth**

**ϕ_cc_: soil moisture at field capacity**

**Lambda cyhalothrin**

| **Soil type** | **ρ_d_** | **f_oc_ %** | **d** | **ϕ_cc_ %hg** | **DF** | **AF** | **AFT** | **INT DF** | **Classification** |
| --- | --- | --- | --- | --- | --- | --- | --- | --- | --- |
| a3s3n4_Ap | 1.2 | 0.1110 | 10 | 57.61 | 417.35 | 5.537.E-10 | 30.7566 | Very immobile | Very unlikely |
| a2s3n3_Ap | 1.07 | 0.0965 | 12 | 62.94 | 296.33 | 2.417.E-09 | 28.6301 | Very immobile | Very unlikely |
| a1s2n2_Aj | 1.18 | 0.1686 | 10 | 57.31 | 625.88 | 1.551.E-14 | 45.8835 | Very immobile | Very unlikely |
| a1s3n2_Aj | 0.82 | 0.1203 | 10 | 58.14 | 306.53 | 1.376.E-07 | 22.7974 | Very immobile | Very unlikely |
| a3s3n4_Bwg | 1.3 | 0.0166 | 45 | 57.61 | 68.30 | 1.523.E-07 | 22.6510 | Very immobile | Very unlikely |
| a2s3n3_Bwg | 1.25 | 0.0343 | 18 | 62.94 | 123.63 | 4.054.E-06 | 17.9161 | Very immobile | Very unlikely |
| a1s2n2_Oaj | 0.67 | 0.3081 | 25 | 57.31 | 649.43 | 1.504.E-36 | 119.0259 | Very immobile | Very unlikely |
| a1s3n2_Oaj | 0.36 | 0.2384 | 27 | 58.14 | 266.68 | 7.640.E-17 | 53.5505 | Very immobile | Very unlikely |
| a3s3n4_bgj | 1.33 | 0.0395 | 25 | 57.61 | 165.05 | 7.052.E-10 | 30.4076 | Very immobile | Very unlikely |
| a2s3n3_Bgj | 1.29 | 0.0340 | 40 | 62.94 | 126.48 | 5.509.E-13 | 40.7319 | Very immobile | Very unlikely |
| a1s2n2_bj | 1.17 | 0.0534 | 25 | 57.31 | 197.13 | 1.338.E-11 | 36.1292 | Very immobile | Very unlikely |
| a1s3n2_Oej | 0.35 | 0.2616 | 13 | 58.14 | 284.50 | 5.266.E-09 | 27.5065 | Very immobile | Very unlikely |
| a3s3n4_oej | 0.3 | 0.2965 | 30 | 57.61 | 278.93 | 2.756.E-19 | 61.6672 | Very immobile | Very unlikely |
| a2s3n3_oej | 0.7 | 0.2093 | 40 | 62.94 | 420.00 | 1.952.E-41 | 135.2625 | Very immobile | Very unlikely |
| a1s2n2_ogj | 0.23 | 0.2616 | 40 | 57.31 | 190.00 | 1.705.E-17 | 55.7152 | Very immobile | Very unlikely |
| a1s3n2_Oij | 0.26 | 0.3721 | 60 | 58.14 | 300.52 | 4.365.E-41 | 134.1016 | Very immobile | Very unlikely |

**Chlorpyrifos**

| **Soil type** | **ρ_d_** | **f_oc_ %** | **d** | **ϕ_cc_ %hg** | **DF** | **AF** | **AFT** | **INT DF** | **Classification** |
| --- | --- | --- | --- | --- | --- | --- | --- | --- | --- |
| a3s3n4_Ap | 1.20 | 0.1110 | 10 | 57.61 | 15.04 | 4.64.E-01 | 1.1083 | Very immobile | Very likely |
| a2s3n3_Ap | 1.07 | 0.0965 | 12 | 62.94 | 10.96 | 4.80.E-01 | 1.0589 | Very immobile | Very likely |
| a1s2n2_Aj | 1.18 | 0.1686 | 10 | 57.31 | 22.07 | 3.26.E-01 | 1.6181 | Very immobile | Very likely |
| a1s3n2_Aj | 0.82 | 0.1203 | 10 | 58.14 | 11.30 | 5.58.E-01 | 0.8406 | Very immobile | Very likely |
| a3s3n4_Bwg | 1.30 | 0.0166 | 45 | 57.61 | 3.27 | 4.72.E-01 | 1.0843 | Moderately immobile | Very likely |
| a2s3n3_Bwg | 1.25 | 0.0343 | 18 | 62.94 | 5.14 | 5.97.E-01 | 0.7442 | Moderately immobile | Very likely |
| a1s2n2_Oaj | 0.67 | 0.3081 | 25 | 57.31 | 22.87 | 5.48.E-02 | 4.1909 | Very immobile | Moderately likely |
| a1s3n2_Oaj | 0.36 | 0.2384 | 27 | 58.14 | 9.96 | 2.50.E-01 | 1.9999 | Moderately immobile | Very likely |
| a3s3n4_bgj | 1.33 | 0.0395 | 25 | 57.61 | 6.53 | 4.34.E-01 | 1.2034 | Moderately immobile | Very likely |
| a2s3n3_Bgj | 1.29 | 0.0340 | 40 | 62.94 | 5.23 | 3.11.E-01 | 1.6848 | Moderately immobile | Very likely |
| a1s2n2_bj | 1.17 | 0.0534 | 25 | 57.31 | 7.61 | 3.80.E-01 | 1.3955 | Moderately immobile | Very likely |
| a1s3n2_Oej | 0.35 | 0.2616 | 13 | 58.14 | 10.56 | 4.93.E-01 | 1.0210 | Very immobile | Very likely |
| a3s3n4_oej | 0.30 | 0.2965 | 30 | 57.61 | 10.37 | 2.04.E-01 | 2.2932 | Very immobile | Likely |
| a2s3n3_oej | 0.70 | 0.2093 | 40 | 62.94 | 15.13 | 3.42.E-02 | 4.8725 | Very immobile | Moderately likely |
| a1s2n2_ogj | 0.23 | 0.2616 | 40 | 57.31 | 7.37 | 2.23.E-01 | 2.1622 | Moderately immobile | Likely |
| a1s3n2_Oij | 0.26 | 0.3721 | 60 | 58.14 | 11.10 | 3.23.E-02 | 4.9534 | Very immobile | Moderately likely |
